# Supplementary material for: Electrochemical DNA hairpin sensors for differentiating small molecule intercalation from minor groove binding
Source: Sens Diagn. 2026 Sep 23;5(11):1383–93. doi: 10.1039/d6sd00111d (PMC13625175; doi:10.1039/d6sd00111d)
Supplement: Supplementary file 2 [file SD-005-D6SD00111D-s001.pdf]

This journal is © The Royal Society of Chemistry 2026

SUPPLEMENTARY INFORMATION

Electrochemical DNA Hairpin Sensors for Differentiating Small Molecule Intercalation from Minor Groove Binding

Kiara Thompson,[1,γ] Kian Barnes[2,γ], Saron Yoseph[3], Vincent Clark[4], J.D. Mahlum[4], Ananya Majumdar[5], Philip S Lukeman[6], Netzahualcóyotl Arroyo-Currás[1,2,3,4,*]

1 Program in Molecular Biophysics, Zanvyl Krieger School of Arts & Sciences, Johns Hopkins University, Baltimore, MD 21218, United States

2 Department of Chemistry, University of North Carolina at Chapel Hill, Chapel Hill, North Carolina 27599, United States

3 Department of Physiology, Pharmacology & Therapeutics, Johns Hopkins School of Medicine, Baltimore, MD 21205, United States

4 Chemistry-Biology Interface Program, Zanvyl Krieger School of Arts & Sciences, Johns Hopkins University, Baltimore, MD 21218, United States

5 Biomolecular NMR Center, Johns Hopkins University, Baltimore, Maryland 21218, United States

6 Department of Chemistry, St. John's University, Queens, NY 11372 USA

γ Denotes equal contribution.

* **Correspondence to:**
Netzahualcóyotl Arroyo-Currás (Netz Arroyo)
Current Address:
Caudill Labs, Room 322
University of North Carolina at Chapel Hill
131 South Road
Chapel Hill, NC 27514
narroyo@unc.edu

Table of Contents

Figure S1: Comparison of sensing buffer peak frequencies.. 3

Figure S2: Jacketed glass cell used for temperature-controlled measurements.......................... 4

Figure S3: Predicted hairpin structures of non-sensing constructs HP8 and HP10. 5

Figure S4: NMR imino proton region of HP4 at multiple temperatures 6

Figure S5: HP4 melting observed by temperature-resolved square wave frequency maps 7

Figure S6: Frequency Map Analysis of Extended Hairpins at Elevated Temperatures 8

Figure S7: Identification of square wave frequencies for optimal hairpin interrogation 9

Figure S8: Control DMSO titration... 10

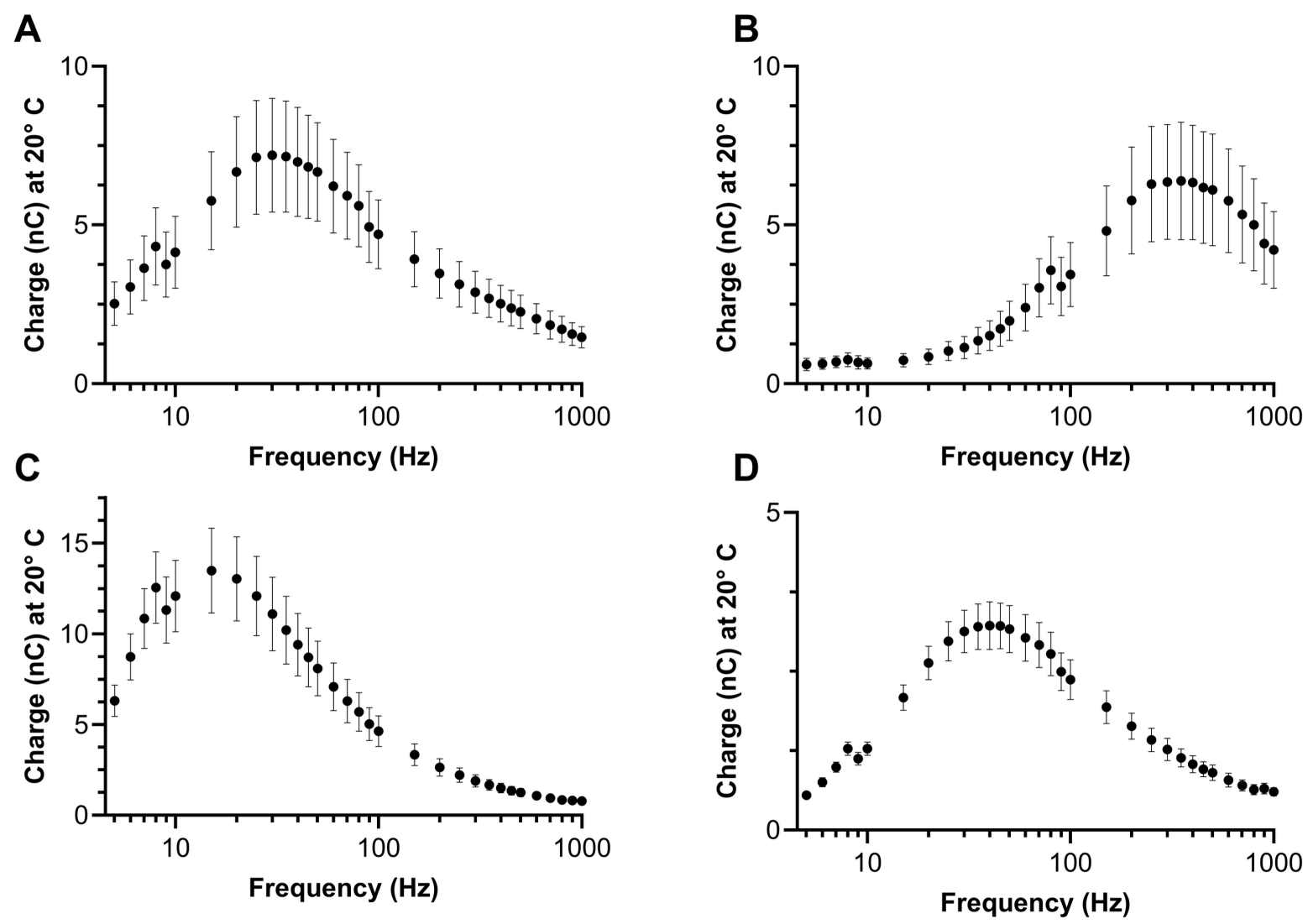

Figure S1: Comparison of sensing buffer peak frequencies

(**A**) Frequency map of HP4 in 100 mM Tris-HCl (pH 7.4) with a peak frequency of 30 Hz. (**B**) Frequency map of HP4 in 100 mM Tris-HCl (pH 7.4) containing 1.25 µM SYBR Gold with a peak frequency of 350 Hz. (**C**) Frequency map of HP4 in 1× PBS (pH 7.4) with a peak frequency of 15 Hz. (**D**) Frequency map of HP4 in 1× PBS (pH 7.4) containing 1.25 µM SYBR Gold with a peak frequency of 40 Hz. Data represent the mean of four independently prepared sensors fabricated using 250 nM HP4; error bars indicate standard deviation.

Tris-HCl was used for frequency-map characterization and dose-response measurements because it produced substantially greater peak-frequency separation than PBS. The increased separation improves visualization of the sensor state transition and enhances the differential signal gain used for quantitative analysis.

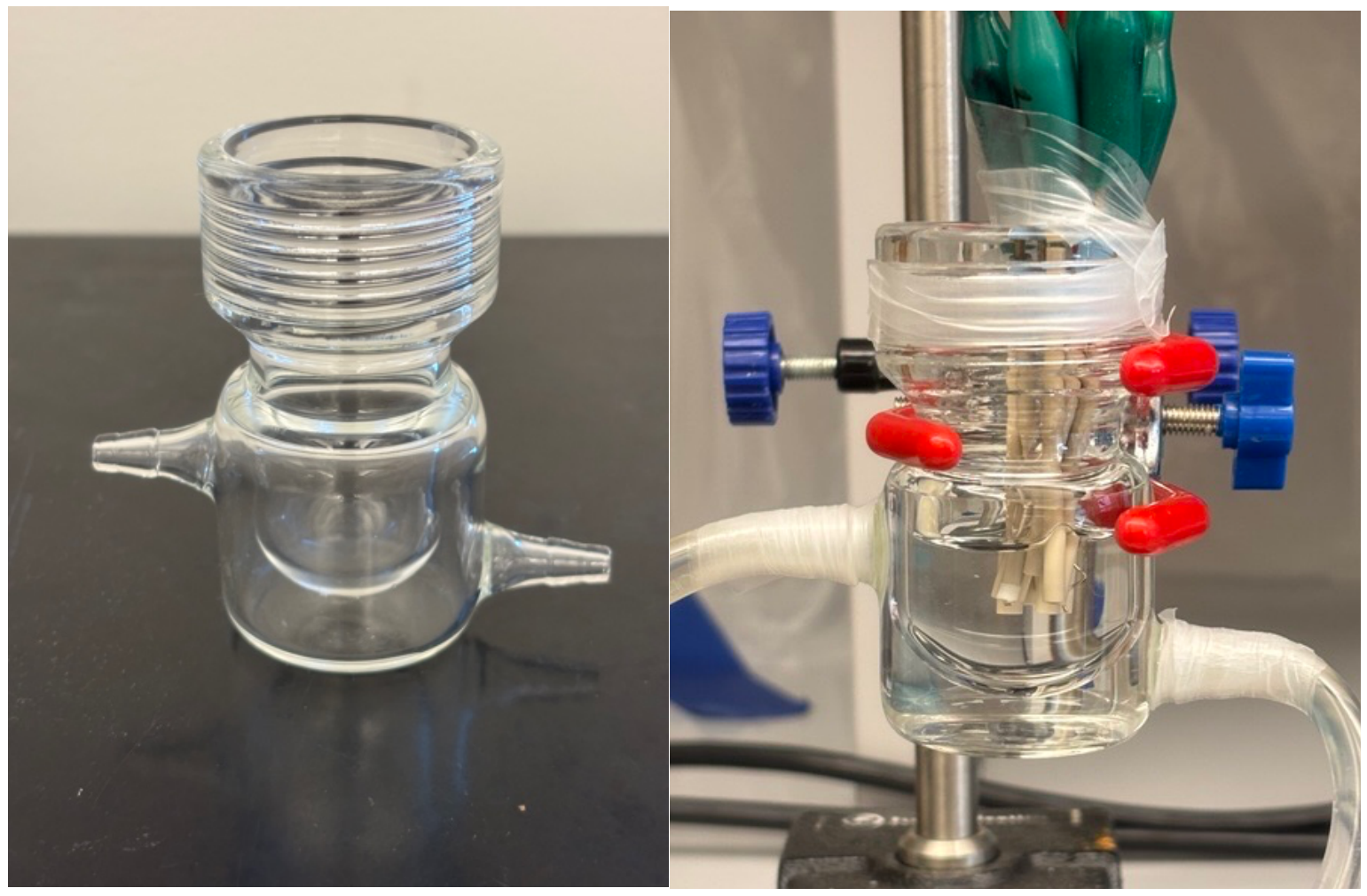

Figure S2: Jacketed glass cell used for temperature-controlled measurements

Left: Empty glass cell. Internal volume is 10 mL. *Right:* Full setup showing water line connections and sensors placed in blank buffered solution.

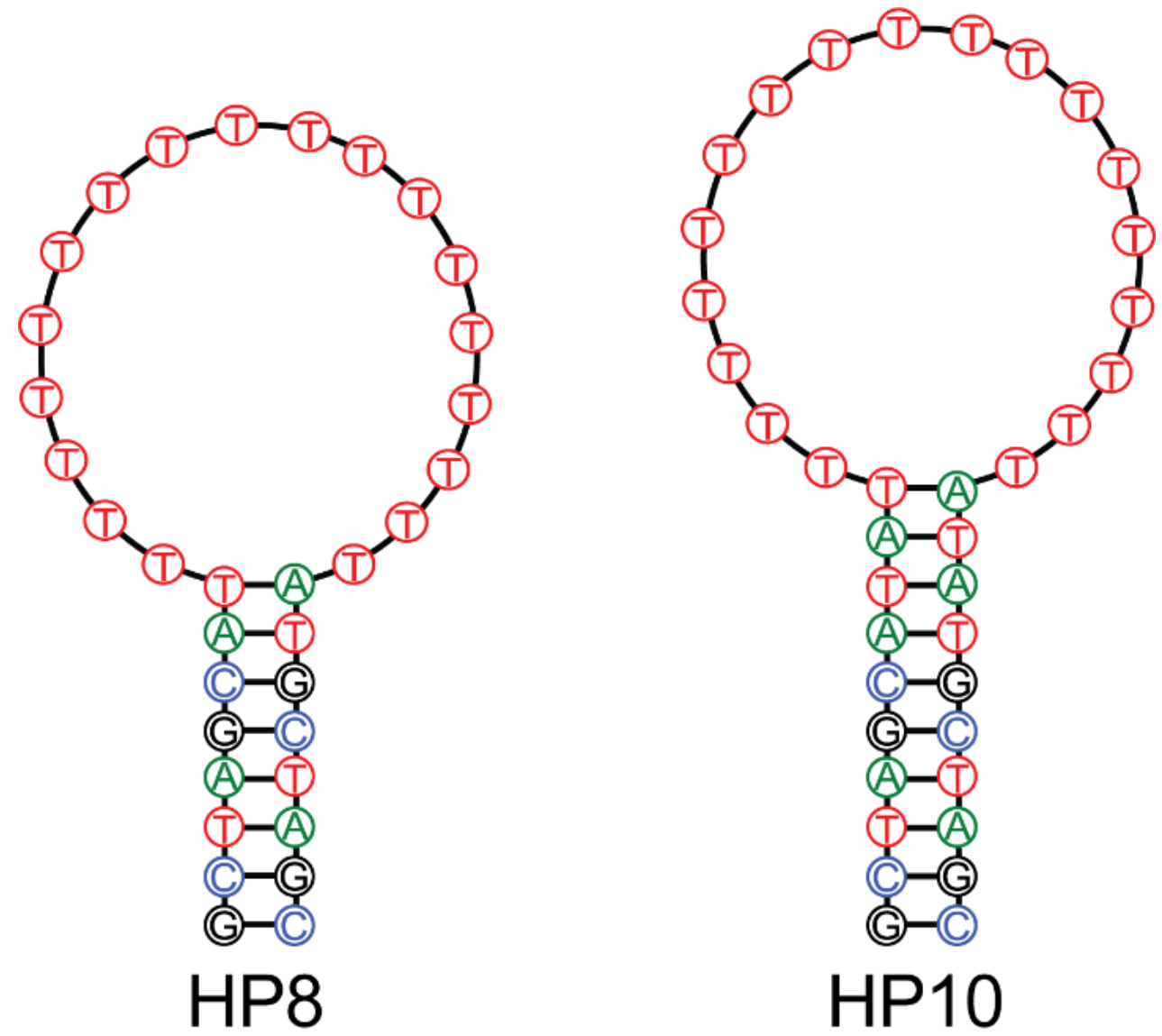

Figure S3: Predicted hairpin structures of non-sensing constructs HP8 and HP10.

NUPACK-predicted secondary structures of HP8 and HP10. These longer-stem constructs were included as controls in solution-phase experiments but were not investigated as nucleic acid-based electrochemical sensors.

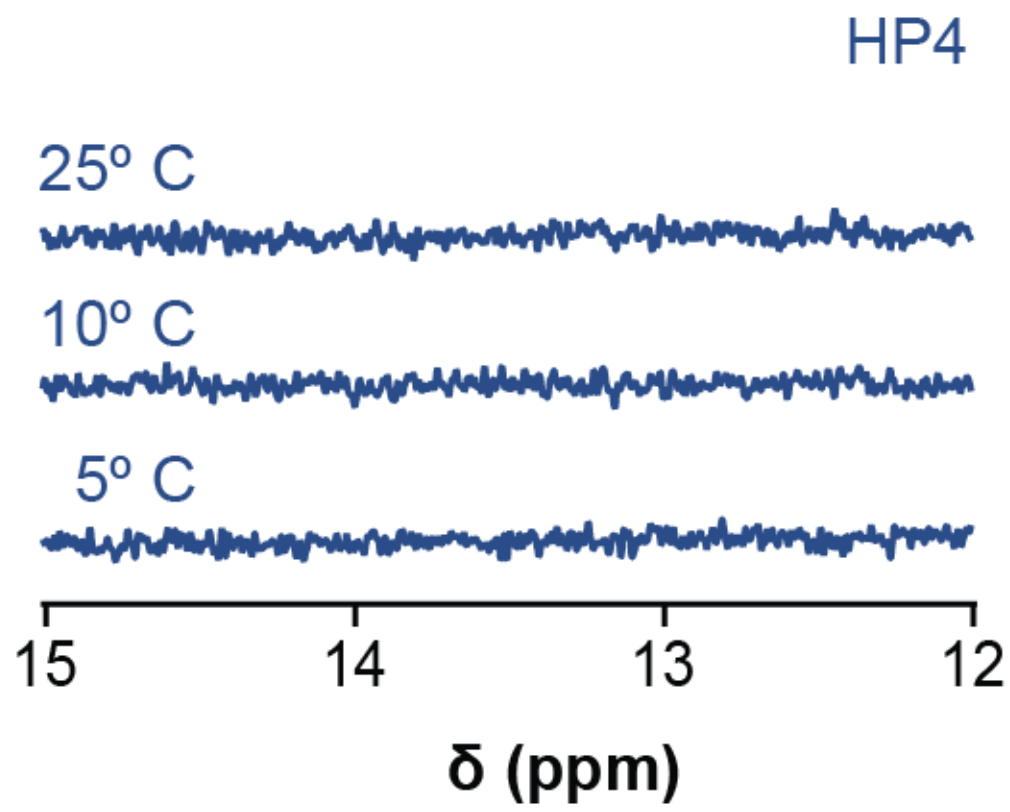

Figure S4: NMR imino proton region of HP4 at multiple temperatures

No observable imino proton resonances were detected for HP4 across the temperatures examined. The absence of signal is consistent with extensive stem fraying, which leaves many imino protons solvent-exposed and susceptible to rapid exchange with water. The resulting exchange broadening renders the imino proton resonances undetectable.

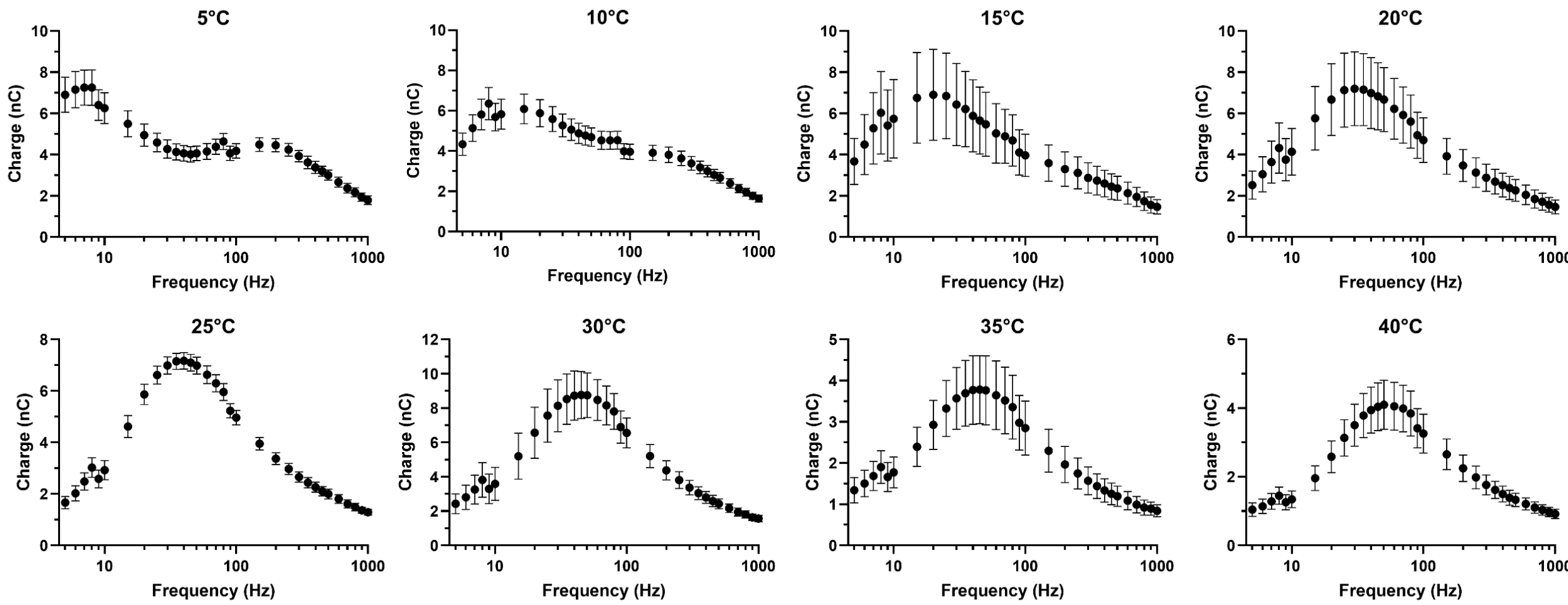

Figure S5: HP4 melting observed by temperature-resolved square wave frequency maps

Frequency maps were measured on HP4 sensors from 5°C to 40°C. Plots show the average of 4 sensors prepared in 250 nM of HP4, errors are the standard deviation. Sensors were independently prepared for each panel. Variability in error is due to small variation in DNA packing densities on the electrode surface due to day-to-day experimental variation.

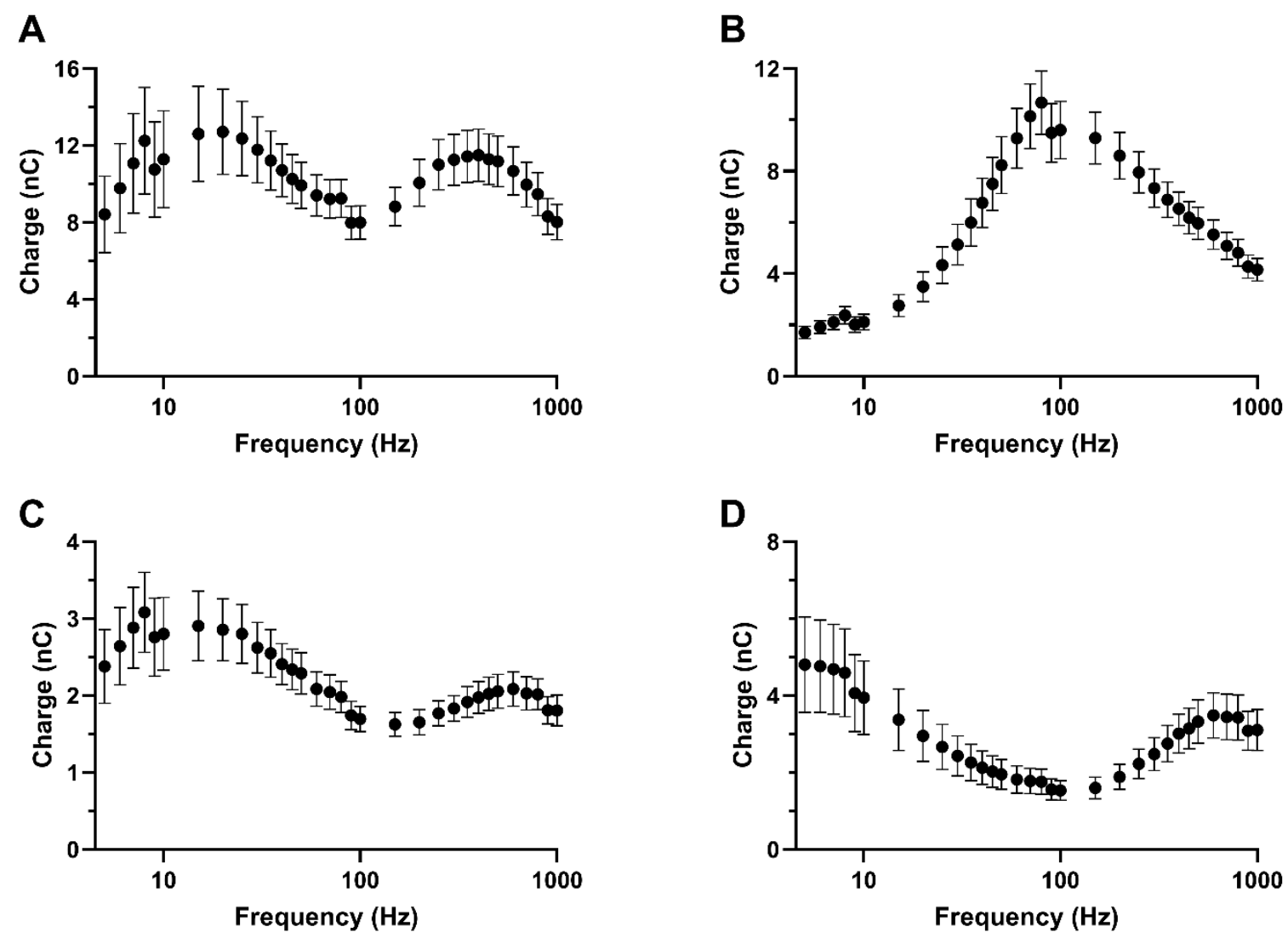

Figure S6: Frequency Map Analysis of Extended Hairpins at Elevated Temperatures

(**A**) Frequency map of HP5 performed at 25°C. (**B**) Frequency map of HP5 performed at 40°C. (**C**) Frequency map of HP6 performed at 40°C. (**D**) Frequency map of HP7 performed at 40°C. All panels show the average and standard deviation of 4 sensors independently prepared for each panel in 250 nM of their respective DNA.

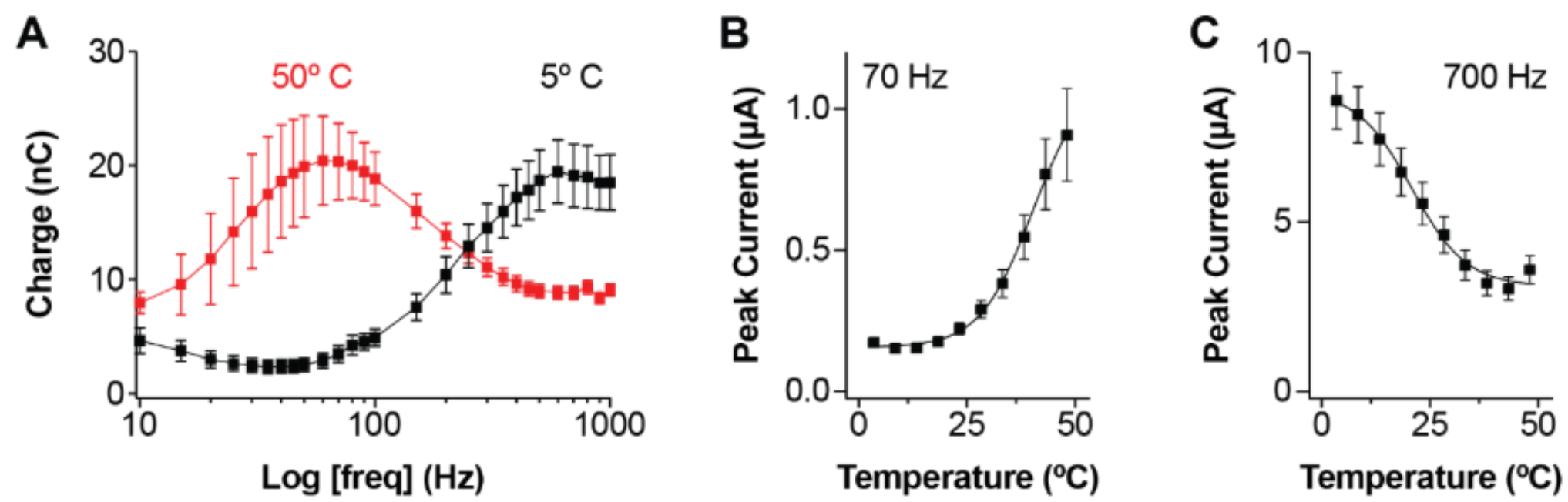

Figure S7: Identification of square wave frequencies for optimal hairpin interrogation

(**A**) To interrogate our NBEs, we first built square wave frequency maps of HP7 to determine best frequencies for interrogating slow and fast electron transferring oligo populations. (**B**) Electrochemical melting measurements of HP**7** conducted at 70 Hz reveal a small, slow electron transferring oligo fractional population that increases with melting. (**C**) Conversely, melting measurements of HP4 conducted at 700 Hz reveal an opposite trend, with a fast electron transferring population that decreases with melting. Square wave amplitude of 50 mV and step size of 1 mV for all measurements. Solid squares represent the mean of 8 independent NBEs, errors represent their standard deviation. Solid lines are point connectors**.**

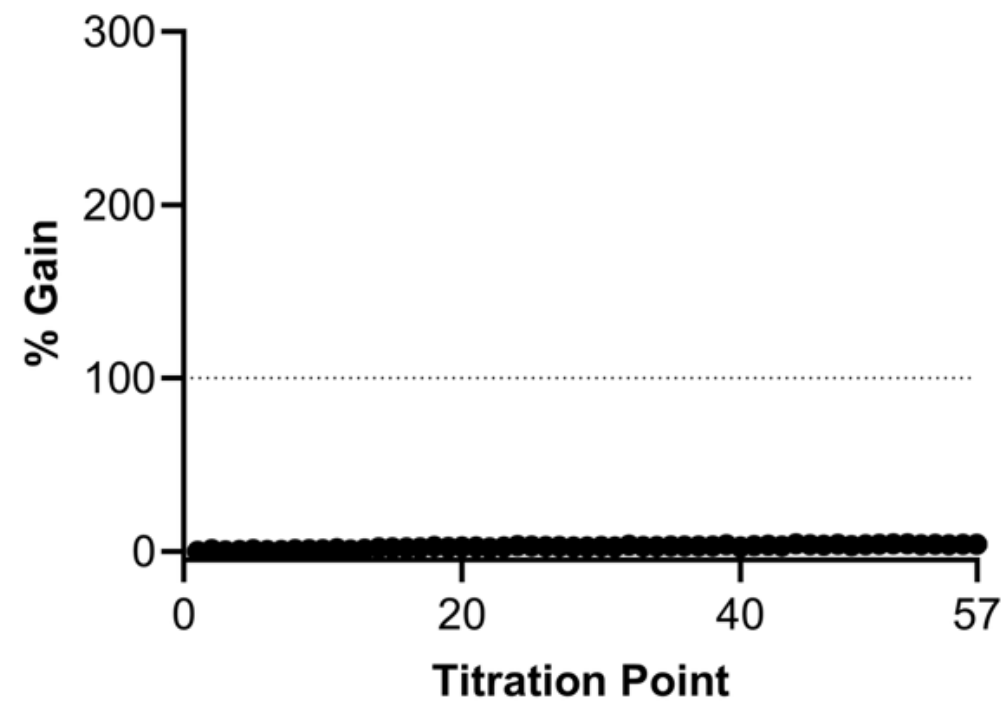

Figure S8: Control DMSO titration

Additions of DMSO in buffered solution in the same volumes and number as those employed for intercalator titrations resulted in negligible signal change for HP4-functionalized sensors.
